# Practical Surgery, with One Hundred and Fifty Engravings on Wood

**Published:** 1842-03-26

**Authors:** 


*Practical Surgery, with one hundred and fifty engravings on wood.* By Robert Liston, Surgeon. Second American from the third London edition. *With additional notes and illustrations.* By George W. Norris, one of the Surgeons of the Pennsylvania Hospital. Philadelphia, 1842. pp. 588. Octavo.

We speak quite within the range of propriety in calling Mr. Liston one of the boldest, ablest, and most successful of the operators that have figured in the ranks of British surgery, and our readers may probably charge us with neglect in not sooner noticing the appearance of a second edition of the above mentioned work.

The delay has been the result of a desire to present a critical analysis of at least, a few of the opinions and processes [advanced or advocated by the author. This design we are compelled to relinquish, because a proper review of a work so crowded with details would greatly exceed the bounds of a weekly journal, and we are therefore obliged to content ourselves with merely introducing it, in a general manner, to the notice of those who have not examined the previous edition.

As no scientific labour is above censure, we will venture upon a little legitimate fault-finding in the first place. The two earliest chapters, comprising thirty-three pages, are devoted to purely elementary directions, in relation to incisions, ligatures, dressings, &c. These are by no means so extensive as would be desirable, and are given occasionally in a sweeping style, which reminds us of the manner of John Bell when denouncing the views of an opponent—a style by no means the most favourable for the conveyance of safe information to mere tyros, for whom alone the directions appear to be intended. The condemnation of greasy dressings and the use of pressure in wounds, for instance, though founded in justice, are liable to be misunderstood and misapplied practically by young practitioners. The too great generality of some of the remarks under censure are no doubt due to the desire that but little space should be devoted to these first lines; but as these chapters are in no degree necessary to the unity or completeness of the volume, we cannot repress the wish either that the author had omitted them altogether, or that the American annotator had extended them so as to include not only a few general laws in relation to minor operations and dressings, but the exceptions also, without a knowledge of which the application of general laws is rarely safe in surgery.

The other chapters, present a wide view of operative surgery as now practised in England, including the novel operations, and illustrated by cases drawn from the very ample experience of the author, though strongly tinctured with peculiar opinions. The disposition of the subjects is chiefly in accordance with the artificial method of classification, arranging the accidents and surgical diseases pretty nearly in the order of the regions, systems, or tissues in which they chance to occur, and not according to the affinities of the morbid conditions. This, which is the great defect of many of the best works on the institutes of surgery, is by no means an objection to a work on operative surgery, like that under notice, and the book will be found one of easy reference. It should be found on the shelves of every surgeon residing at a distance from large libraries, who wishes to be familiar with the existing condition of his art in England.

The present edition is considerably improved upon its predecessor, and is published in a style which does great credit to the imprint of Thomas, Cowperthwait & Co.

Some very valuable matter has been added by the American editor,—we regret that he has not given us more; it would have improved the work. R. C.

